# BMP9-Induced Survival Effect in Liver Tumor Cells Requires p38MAPK Activation

**DOI:** 10.3390/ijms160920431

**Published:** 2015-08-28

**Authors:** María García-Álvaro, Annalisa Addante, Cesáreo Roncero, Margarita Fernández, Isabel Fabregat, Aránzazu Sánchez, Blanca Herrera

**Affiliations:** 1 Department of Biochemistry and Molecular Biology II, Faculty of Pharmacy, Complutense University of Madrid. San Carlos Clinical Hospital Health Research Institute (IdISSC), Plaza Ramón y Cajal S/N, Madrid 28040, Spain; E-Mails: mariagarciaalvaro@gmail.com (M.G.-A.); aaddante@ucm.es (A.A.); ceronce@ucm.es (C.R.); margafdz@ucm.es (M.F.); munozas@ucm.es (A.S.); 2 Bellvitge Biomedical Research Institute (IDIBELL) and University of Barcelona (UB), L’Hospitalet de Llobregat, Barcelona 08908, Spain; E-Mail: ifabregat@idibell.cat

**Keywords:** BMP9, non-Smad, apoptosis, liver cancer, p38MAPK, PI3K

## Abstract

The study of bone morphogenetic proteins (BMPs) role in tumorigenic processes, and specifically in the liver, has gathered importance in the last few years. Previous studies have shown that BMP9 is overexpressed in about 40% of hepatocellular carcinoma (HCC) patients. *In vitro* data have also shown evidence that BMP9 has a pro-tumorigenic action, not only by inducing epithelial to mesenchymal transition (EMT) and migration, but also by promoting proliferation and survival in liver cancer cells. However, the precise mechanisms driving these effects have not yet been established. In the present work, we deepened our studies into the intracellular mechanisms implicated in the BMP9 proliferative and pro-survival effect on liver tumor cells. In HepG2 cells, BMP9 induces both Smad and non-Smad signaling cascades, specifically PI3K/AKT and p38MAPK. However, only the p38MAPK pathway contributes to the BMP9 growth-promoting effect on these cells. Using genetic and pharmacological approaches, we demonstrate that p38MAPK activation, although dispensable for the BMP9 proliferative activity, is required for the BMP9 protective effect on serum withdrawal-induced apoptosis. These findings contribute to a better understanding of the signaling pathways involved in the BMP9 pro-tumorigenic role in liver tumor cells.

## 1. Introduction

Unlike most solid tumors, the incidence and mortality of hepatocellular carcinoma (HCC) has increased in the last decade, having an enormous socio-economic cost. HCC ranks as the sixth most common cancer and results in more than 600,000 deaths per annum worldwide [1,2]. One of the reasons behind this reality is the lack of effective therapies, in part due to the fact that molecular mechanisms of hepatocarcinogenesis are only partly understood. In this regard, a more comprehensive understanding of the cascade of molecular and cellular reactions involved in HCC initiation and progression will undoubtedly help to find new pharmacological targets.

Bone morphogenetic proteins (BMPs) are a large subfamily included in the TGF-β superfamily of cytokines. Although originally discovered for their capacity to induce bone formation [3,4], it has become clear that the role of BMPs is not restricted to this tissue. It is well established that BMP signaling has a key role in early liver development [5,6], and recently, also a role of BMPs in adult liver homeostasis has started to be unveiled. In fact, BMPs are involved in several aspects of liver physiology and pathology, including hepatic fibrosis and regeneration [7]. In particular, the role that BMPs plays in liver cancer has only started to be studied in the past few years. Evidence supports a pro-tumorigenic action of BMPs in HCC: thus, *in vitro* data indicate that BMP4 regulates migration, invasion and anchorage-dependent and -independent growth of HCC cell lines [8,9]. These results are further supported by data obtained with BMP antagonists: incubation with noggin and chordin diminished HCC cell invasion and migration, therefore confirming the involvement of BMP signaling in these processes in liver cancer cells [10]. In line with this, BMP4 has been shown to be overexpressed in cirrhosis and HCC [8,11] and associated with poor prognosis in HCC [12]. The role of other BMP family members is unclear, although new evidence also reveals that BMP7 and BMP6 are overexpressed in different liver cancer models, such as hepatitis B virus X antigen transgenic mouse [10,11].

To add further complication to this scenario, BMP9 has also been related to hepatocarcinogenic processes. BMP9 is expressed in healthy liver [13,14], but overexpressed in a subset of human HCC tissues and cell lines, as shown by our and other laboratories [10,15,16]. In transformed hepatic cells, BMP9 elicits an epithelial to mesenchymal transition (EMT) process that increases cell migration [16]. In the same line of evidence, our previous work indicates that HCC cells present an autocrine production of BMP9 that increases cell growth. Specifically, we have demonstrated that BMP9 increases cell proliferation and impairs low serum-triggered apoptosis in the liver tumor cell line HepG2 [15], although molecular mechanisms driving these effects were not determined.

BMP9 binds to a heterotetrameric transmembrane receptor complex formed by specific type I and type II serine/threonine kinase receptors. Once the receptor complex is activated, it recruits and phosphorylates the R-Smads, Smad1,5,8 that bind to Smad4 to translocate to the nucleus and modulate gene expression. Importantly, in certain cellular types, BMP9 and other BMP ligands also activate other signaling pathways, known as non-canonical or non-Smad signaling pathways. In fact, although it is clear that some of the biological actions exerted by BMPs are mediated by non-Smad intracellular mechanisms [17], the specific contribution of those to BMP9 cellular functions is only partly understood.

Here, we have studied what signaling pathways drive BMP9’s effects in liver tumor cells and found that BMP9 induces canonical and non-canonical signaling pathways, specifically PI3K/AKT and p38MAPK cascades. Our data have revealed that the PI3K/AKT pathway is not involved in the BMP9 growth effect in these cells and that p38MAPK activation is required for the BMP9 survival effect against serum deprivation-induced apoptosis.

## 2. Results

### 2.1. BMP9 Promotes HepG2 Cell Growth through Cell Cycle Regulation and Survival

**Figure 1:**
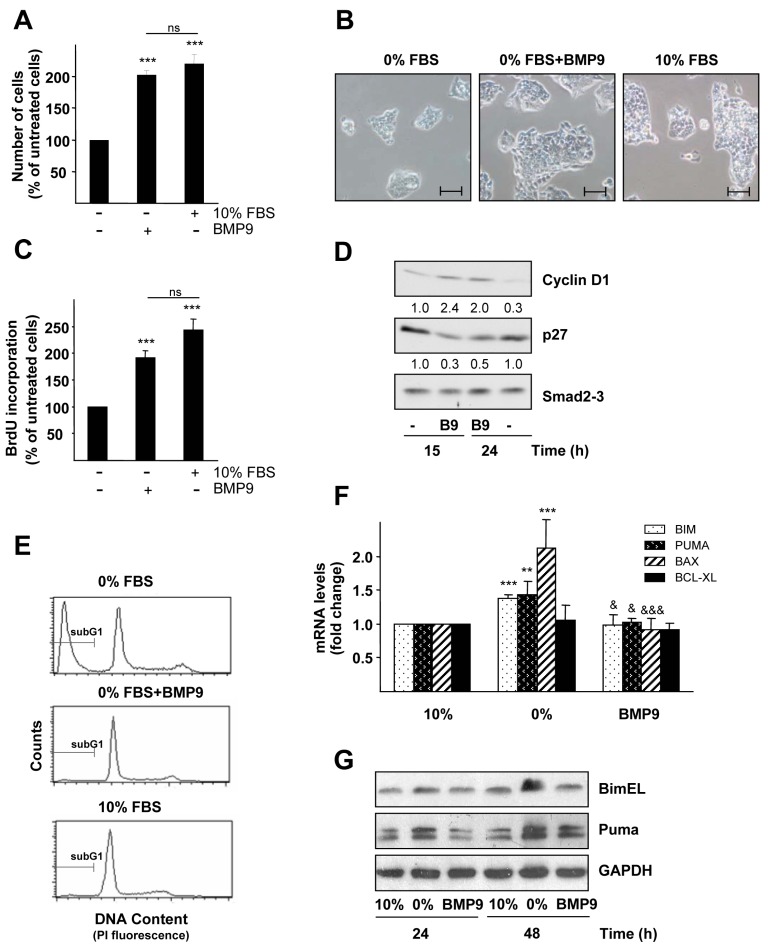
BMP9 increases HepG2 cell number: effects on cell cycle and survival. (**A**) HepG2 cells were incubated in 10% FBS media or in 0% FBS media with or without BMP9 (5 ng/mL) for four days, and then, the cell number was analyzed by crystal violet staining. Data from two independent experiments with *n =* 6 displayed as the percentage of 0% FBS-treated cells (mean ± S.E.M.). Statistical analysis was carried out using the paired *t*-test, and data were compared to untreated cells (0% FBS samples), *** *p* < 0.001 or compared as indicated. n.s., not significant; (**B**) HepG2 cells were incubated in 10% FBS media or in 0% FBS media with or without BMP9 (5 ng/mL) for three days. Cells were visualized, and pictures were taken by phase contrast microscopy. Scale bars represent 50 μm; (**C**) HepG2 cells were incubated in 10% FBS media or in 0% FBS media with or without BMP9 (5 ng/mL) for two days, and proliferation by BrdU incorporation was analyzed. Data from one representative experiment out of four, with *n =* 6, displayed as the percentage of untreated cells (0% FBS samples) (mean ± S.D.). Statistical analysis was carried out as in (**A**); (**D**) HepG2 cells were incubated for different periods of time −/+ BMP9 (5 ng/mL) in 0% FBS media. Western blots were performed with antibodies that recognize cyclin D1, p27 and Smad2,3 analyzed as the loading control. Optical density values relative to the loading control were calculated. A representative experiment of two is shown; (**E**) HepG2 cells were incubated in 10% FBS media or in 0% FBS media −/+ BMP9 (5 ng/mL) for four days, and then, nuclear DNA content was analyzed by flow cytometry. SubG1 cells are hypodiploid cells, cells with a DNA content lower than 2n. Data from one representative out of four experiments, with *n =* 2, are shown; (**F**) HepG2 cells were incubated in 10% FBS media or in 0% FBS media −/+ BMP9 (5 ng/mL) for 24 h and Bim, Puma, Bax and Bcl-xL mRNA levels were analyzed by RT-qPCR and normalized to Gusb. Fold changes relative to 10% FBS media samples were determined (mean ± S.E.M., *n =* 4). Statistical analysis was carried out using the paired *t*-test, and data were compared to 10% FBS samples, ** *p* < 0.01, *** *p* < 0.001 or compared to 0% FBS samples, ^&^ *p* < 0.05, ^&&&^ *p* < 0.001; (**G**) HepG2 cells were incubated −/+ BMP9 (5 ng/mL) in 0% FBS media or in the presence of 10% FBS media for different periods of time. Western blots were performed with antibodies that recognize Bim, Puma and GAPDH (loading control). A representative experiment of three is shown.

We have previously described that BMP9 is a strong mitogen for liver tumor cells in the presence of 0.1% FBS [15]. Our current study also shows this effect in the absence of serum. In fact, when HepG2 cells were incubated with BMP9 for four days in 0% FBS, we found that the number of viable adherent cells doubled in comparison to untreated cells. Indeed, BMP9 treatment in the absence of serum resulted in cell growth rates similar to those observed in the presence of 10% FBS (normal growing conditions). Furthermore, the BMP9 cell growth effect was readily visible by phase contrast microscopy (Figure 1A,B). Consistently, BMP9 induces an increase in BrdU incorporation to nearly the same extent as that obtained when cells were incubated in 10% FBS (Figure 1C). Increased cell proliferation induced by BMP9 was accompanied by changes in the expression of cell cycle regulators: BMP9 enhanced cyclinD1 expression and decreased CDK interacting protein/kinase inhibitory protein p27 expression (Figure 1D), both events involved in the progression from the G0/G1 phases towards the S phase of the cell cycle [18]. We had documented before that incubation of HepG2 cells in low serum, 0.1% FBS, resulted in an apoptotic cell death that was rescued by BMP9 [15]. Data presented here indicate that the BMP9 pro-survival effect is also observed when cells are incubated in the complete absence of serum (Figure 1E). It is well established that serum deprivation in HCC cells results in a mitochondrial apoptosis characterized by mitochondrial membrane potential depletion, cytochrome c release and Bcl-2 family member modulation [19,20,21]. Our results are in agreement with these previous results, as we observed that serum starvation resulted in the upregulation of several pro-apoptotic members of the Bcl-2 family, in particular Bim, Puma and Bax, both at the mRNA and the protein level. Importantly, BMP9 treatment blocked these effects (Figure 1F,G). In parallel, we analyzed the antiapoptotic protein Bcl-xL, but no modulation was observed in any of the conditions tested (Figure 1G).

Altogether, our data confirm that BMP9 plays a significant role in HepG2 cell growth, promoting cell proliferation and survival.

### 2.2. BMP9 Activates Non-Canonical Pathways in Hepg2 Cells

**Figure 2:**
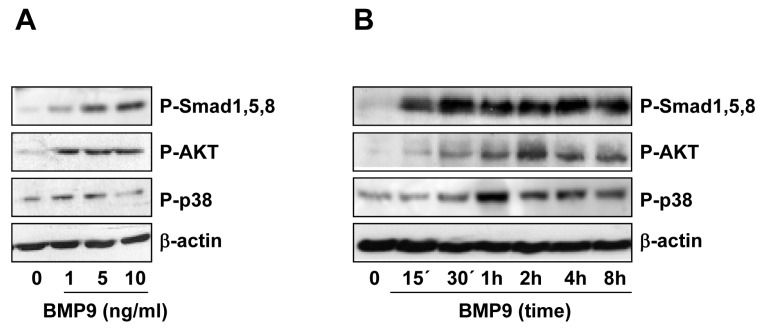
BMP9 activates both canonical and non-canonical pathways in HepG2 cells. HepG2 cells were incubated for one hour with different concentrations of BMP9 (1–10 ng/mL) in 0% FBS media (**A**) or for different periods of time −/+ BMP9 (5 ng/mL) in 0% FBS media (**B**). Western blots were performed with antibodies that recognize phospho-Smad1,5,8, phospho-AKT, phospho-p38 and β-actin (loading control). A representative experiment of two is shown.

BMP9 dose-dependently activates the Smad1,5,8 pathway in HepG2 cells (Figure 2A). Smad1,5,8 phosphorylation was observed after 15 min of BMP9 treatment and sustained up to eight hours (Figure 2B). Since it has been previously shown that in endothelial cells, BMP9 triggers Smad2,3 phosphorylation, the classic TGF-β downstream pathway [22,23,24], we tested this possibility in HepG2 cells. However, we could not detect Smad2 phosphorylation at the time points analyzed (Figure S1A). To investigate whether BMP9 could trigger other non-Smad signaling pathways, we performed Western blotting experiments using antibodies against the phosphorylated (active) forms of MAPK (ERK, p38 and JNK) and AKT, as a read-out of PI3K activation. We could not observe JNK activation in response to BMP9 (data not shown), nor any modulation of phospho-ERK levels (Figure S1B), which are intrinsically elevated in basal conditions in HepG2 cells, as previously reported [25]. On the other hand, BMP9 triggered both AKT and p38MAPK phosphorylation in HepG2 cells (Figure 2A,B). Furthermore, our data showed that the p38MAPK downstream target ATF2 was also phosphorylated upon BMP9 treatment (Figure S1C).

Altogether, these results seem to indicate that BMP9 triggers not only the Smad1,5,8 pathway, but also non-Smad p38MAPK and PI3K/AKT signaling cascades in HepG2 cells.

**Figure 3:**
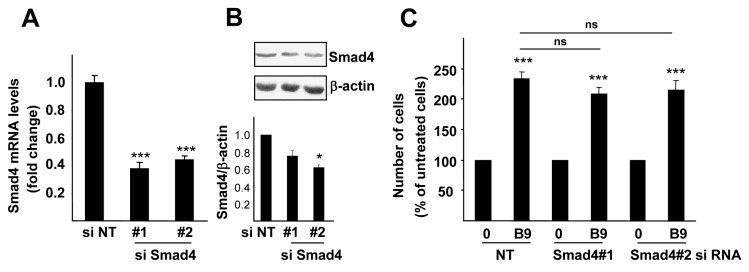
Smad4 knockdown does not impair the BMP9-mediated growth effect in HepG2 cells. HepG2 cells were transiently transfected with non-targeting negative control siRNA (siRNA NT) or with two different Smad4 targeting siRNA (siRNA Smad4 #1 and #2). (**A**) Smad4 RNA levels were determined by RT-qPCR and normalized to β-actin. Data are expressed relative to siRNA NT (assigned an arbitrary value of one), from two different experiments (mean ± S.E.M.); (**B**) Smad4 protein levels were analyzed by Western blot performed with antibodies that recognize Smad4 and β-actin as the loading control (**upper** panel). A representative experiment of three is shown. Optical density values relative to loading control were calculated and expressed as fold change relative to siRNA NT, assigned an arbitrary value of one (**bottom** panel); (**C**) Cells were serum starved and incubated −/+ BMP9 (5 ng/mL) for four days and then counted. Data from two experiments (*n =* 3) are displayed as the percentage of untreated cells (mean ± S.E.M.). Statistical analysis was carried out using the paired *t*-test, and data were compared to untreated samples, * *p* < 0.05, *** *p* < 0.001 or as indicated. n.s., not significant.

### 2.3. p38MAPK, but Not PI3K Activation, Is Required for the BMP9 Growth Promoting Effect in HepG2 Cells

To analyze whether Smad activation was involved in the effect of BMP9 on HepG2 cells, we performed transient knockdown of Smad4 using two different siRNAs obtaining approximately 60% reduction of Smad4 levels. Under these conditions, although the transcription of target genes, such as Hamp or Id1, was severely diminished, BMP9-induced cell growth remained unaffected (Figure 3 and Figure S2). As these data suggested that Smad signaling was not required for the BMP9 growth effect in HepG2 cells, we hypothesized that non-canonical signaling could mediate this effect.

**Figure 4:**
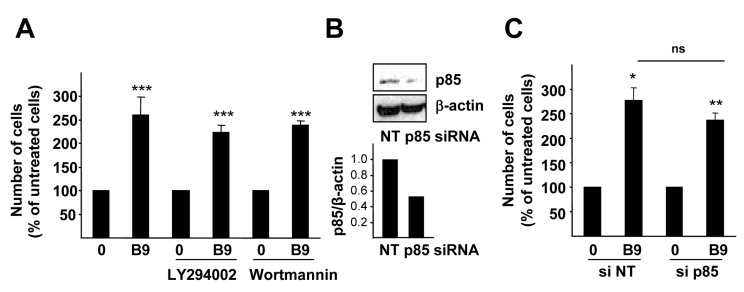
PI3K activity is not required for the BMP9 growth effect in HepG2 cells. (**A**) HepG2 cells were treated with LY294002 (7.5 µM) or Wortmannin (80 nM) (one hour pre-incubation) and −/+ BMP9 (5 ng/mL) in 0% FBS media and counted at day 4. Data from three independent experiments performed in triplicate, displayed as the percentage of untreated cells (mean ± S.E.M.); (**B**,**C**) HepG2 cells were transfected with non-targeting negative control siRNA (siRNA NT) or p85 targeting siRNA (siRNA p85). (**B**) Whole cell lysates were collected and Western blot with anti-p85 antibody was performed. β-Actin was analyzed as the loading control. A representative experiment of two is shown (**upper** panel). Optical density values relative to the loading control were calculated and expressed as the fold change relative to siRNA NT, assigned an arbitrary value of one (**bottom** panel); (**C**) Cells were serum starved and incubated −/+ BMP9 (5 ng/mL) for four days and then counted. Data from one representative experiment (*n =* 4) out of two are shown and are displayed as the percentage of untreated cells (mean ± S.D.). Statistical analysis was carried out using the paired *t*-test, and data were compared to untreated samples, * *p* < 0.05, ** *p* < 0.01, *** *p* < 0.001 or as indicated. n.s., not significant.

Thus, we focused our attention on the PI3K/AKT pathway, since it has been long implicated in cell survival and proliferation [26,27,28]. When cells were pre-incubated with PI3K inhibitors (LY249002 and wortmannin), the BMP9-driven increase in HepG2 cell number was still observed, suggesting that PI3K activation was not required for the BMP9-mediated growth effect (Figure 4A). To further confirm this data, HepG2 cells were transfected with siRNA targeting p85α, the PI3K regulatory subunit, obtaining a 50% decrease. Reduction of p85α expression did not alter BMP9 response in HepG2 cells: BMP9 still induced an increase in cell number (Figure 4B,C).

**Figure 5:**
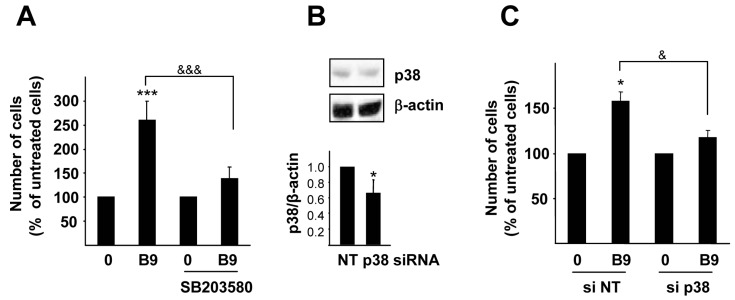
p38MAPK is required for BMP9 growth promoting effect in HepG2 cells. (**A**) HepG2 cells were treated with SB203580 (10 µM) (one hour pre-incubation) and −/+ BMP9 (5 ng/mL) in 0% FBS media and counted at day 4. Data from four independent experiments with *n =* 4, displayed as the percentage of untreated cells (mean ± S.E.M.); (**B**,**C**) HepG2 cells were transfected with non-targeting negative control siRNA (siRNA NT) or p38 targeting siRNA (siRNA p38). (**B**) Whole cell lysates were collected and Western blot with anti-p38 antibody was performed. β-Actin was analyzed as the loading control. A representative experiment of three is shown (**upper** panel). Optical density values relative to the loading control were calculated and expressed as the fold change relative to siRNA NT, assigned an arbitrary value of one (**bottom** panel); (**C**) Cells were serum starved and incubated −/+ BMP9 (5 ng/mL) for four days and then counted. Data from one representative experiment (*n =* 3) out of three are shown, displayed as the percentage of untreated cells (mean ± S.D.). Statistical analysis was carried out using the paired *t*-test, and data were compared to untreated samples, * *p* < 0.05, *** *p* < 0.001 or as indicated, ^&^ *p* < 0.05, ^&&&^ *p* < 0.001.

We then investigated the role of p38MAPK in the BMP9-induced growth effect in HepG2 cells. Pre-incubation with a selective p38MAPK inhibitor SB203580 almost completely abrogated the increase in HepG2 cell number induced by BMP9 (Figure 5A). The specificity of this effect was subsequently proven by cell transfection with siRNA targeting p38MAPK. Although only a silencing efficiency of 30% was obtained, that was sufficient to inhibit the cell growth response to BMP9 (Figure 5B,C).

Taken together, these results suggest that p38MAPK, but not PI3K activity is required for the BMP9 growth effect in HepG2 cells.

### 2.4. p38MAPK Is Required for the BMP9 Survival Effect on Serum Starvation-Triggered Apoptosis in HepG2 Cells

**Figure 6:**
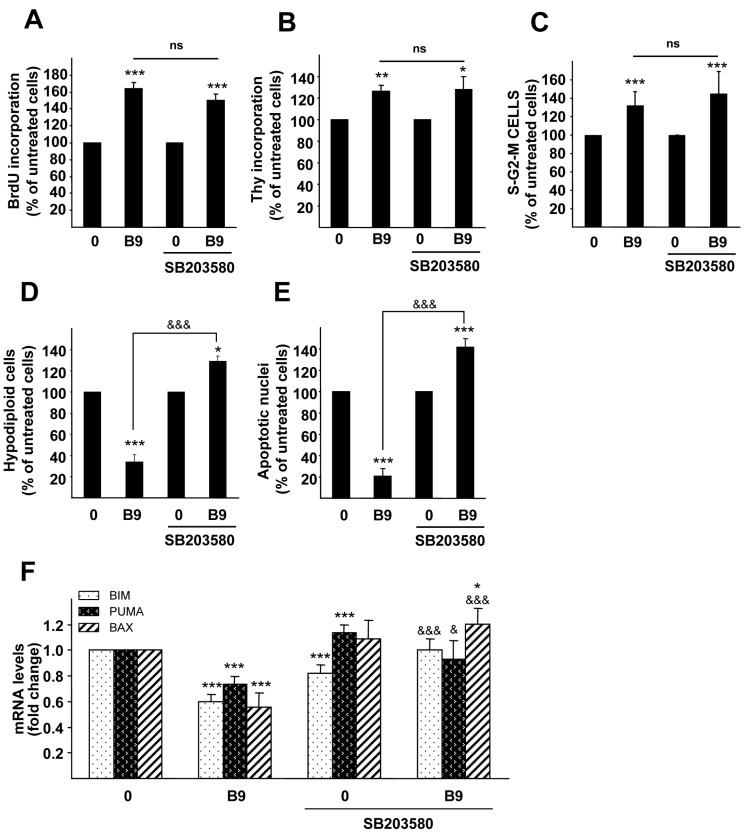
p38MAPK is involved in the BMP9 survival effect on serum starvation-triggered apoptosis in HepG2 cells. (**A**–**C**) HepG2 cells were treated with SB203580 (10 µM) (one hour pre-incubation) and −/+ BMP9 (5 ng/mL) in 0% FBS media for 24 h (**A**) BrdU incorporation assay. Data are the mean ± S.E.M. of four independent experiments and are displayed as the percentage of untreated cells; (**B**) Thymidine incorporation assay. Data are the mean ± S.E.M. of two independent experiments (*n =* 4) and are displayed as the percentage of untreated cells; (**C**) DNA content analyzed by flow cytometry. Percentages of cells corresponding to the S-G2-M phase are shown. Data are the mean ± S.E.M. of three independent experiments (*n =* 3) and are displayed as the percentage of untreated cells; (**D**,**E**) HepG2 cells were treated with SB203580 (10 µM) (one hour pre-incubation) and −/+ BMP9 (5 ng/mL) in 0% FBS media for 72 h. (**D**) Nuclear DNA content was analyzed by flow cytometry, and the percentages of hypodiploid (apoptotic) cells were obtained. Data are from three independent experiments performed in triplicate (mean ± S.E.M.), displayed as the percentage of untreated cells; (**E**) Apoptotic nuclei were visualized and counted after PI staining under a fluorescence microscope. A minimum of 1000 nuclei were counted per condition. Data from two independent experiments performed in triplicate (mean ± S.E.M.) displayed as the percentage of untreated cells. Statistical analysis was carried out using the paired *t*-test, and data were compared to 0% FBS samples, * *p* < 0.05, ** *p* < 0.01, *** *p* < 0.001 or as indicated, ^&&&^ *p* < 0.001. n.s., not significant; (**F**) HepG2 cells were treated with SB203580 (10 µM) (one hour pre-incubation) and −/+ BMP9 (5 ng/mL) in 0% FBS media for 24 h, and Bim, Puma and Bax levels were analyzed by RT-qPCR and normalized to Gusb. Fold changes relative to 0% FBS media samples were determined (mean ± S.E.M., *n =* 4). Statistical analysis was carried out using the paired *t*-test, and data were compared to 0% FBS samples, * *p* < 0.05, *** *p* < 0.001. A *t*-test was also performed between BMP9 and SB203580 + BMP9 samples: ^&^ *p* < 0.05, ^&&&^ *p* < 0.001.

To further analyze the role of p38MAPK in BMP9-triggered responses, we carried out experiments to determine the effect of p38MAPK inhibition on BMP9-mediated proliferation and survival in HepG2 cells. Thus, in the presence of SB203580, the BMP9 effect on proliferation was still observed, determined by BrdU or [3H]-thymidine incorporation assays (Figure 6A,B, respectively) or by flow cytometric analysis of DNA content (Figure 6C). These data suggest that p38MAPK is not involved in the proliferative effect of BMP9 in HepG2 cells. Contrarily, p38MAPK inhibition completely impaired the BMP9-mediated survival effect against serum starvation-induced apoptosis (Figure 6D,E). Furthermore, p38MAPK inhibition abolished BMP9 regulation of Bim, Puma and Bax mRNA levels (Figure 6F), demonstrating that p38MAPK mediates BMP9-dependent survival activity on serum starvation-triggered apoptosis in HepG2 cells.

## 3. Discussion

BMP9 regulates proliferative responses, being able to both inhibit and promote cell growth depending on the cell type. Specifically, in liver cells, BMP9’s effects depend on the transformation and developmental status of cells. Hence, these effects range from a mild cell death observed in adult mouse hepatocytes to a positive cell growth effect in HCC cells, including non-responsive cells, such as human adult hepatocytes and mouse neonatal hepatocytes [15]. The reasons behind these differential cellular responses to the same stimulus are not known. In an effort to understand the intracellular mechanisms that drive BMP9’s effects, we have analyzed the signaling pathways activated by BMP9 in liver cancer cells. Our results clearly demonstrated that in HepG2 cells, BMP9 induces Smad1,5,8, but not Smad2 phosphorylation. Strikingly, Smad signaling does not appear to be involved in the growth effect triggered by BMP9. These results are different from those we found in ovarian cancer cells, namely in OVCA433 cells, Smad1 or Smad4 silencing abrogated BMP9 proliferative effect [29]. This apparent discrepancy highlights once more the complexity of BMP9 signaling and responses, which are cell-type, status and context dependent.

We have focused our efforts in delineating the role of the non-canonical pathways in BMP9 effects on liver cancer cells. In fact, although it is clear that some of the biological actions exerted by the BMPs are mediated by non-Smad intracellular mechanisms [17], the specific contribution of those to BMP9-mediated biological activities is only partly understood. Thus, we have demonstrated that BMP9 activates the PI3K/AKT cascade in HCC cells, although this activation is not required for the BMP9-mediated growth effect. As the PI3K/AKT pathway has been involved in EMT processes in the liver cells [30,31] and BMP9 induces this phenotypic conversion in HCC cells [16], it is tempting to speculate that BMP9-triggered AKT activation could mediate EMT in HepG2 cells, although further studies are necessary to test this hypothesis.

Our results show that BMP9 activates p38MAPK in HepG2 cells; however, how this activation occurs and whether it is a direct or an indirect activation remains to be investigated. BMP9 has already been shown to induce p38MAPK activation in other cellular models, such as lung microvascular endothelial cells, dental follicle stem cells, mesenchymal progenitor cells (MPCs) and osteosarcoma cells [23,32,33,34,35]. Of note, the specific role of p38MAPK activation in the biological activities of BMP9 has not been clearly established. Thus, p38MAPK activity does not contribute to osteogenic marker Osx regulation induced by BMP9 in osteosarcoma cells [33]. An even more complex scenario is found in MPCs. In these cells, BMP9 activates p38MAPK, which mediates MPC proliferation and osteogenic differentiation. Given the fact that p38MAPK interferes with the Smad1,5,8 pathway, it is not clear whether effects observed upon p38MAPK inhibition are due to Smad signaling modulation or due to a direct effect of p38MAPK in osteogenic differentiation [34,35]. In the present study, we have shown that p38MAPK activity is required for BMP9-mediated cell growth in HCC cells, more precisely, for its pro-survival activity against serum withdrawal-induced apoptosis. p38MAPK has been classically associated with stress responses leading to growth inhibition and apoptosis. Nevertheless, in other circumstances, such as in cancerous cells, p38MAPK activation may produce the opposite effect, activating protective pathways [36]. Thus, p38MAPK has been shown to be involved in a protective pathway in DNA-damaged fibroblasts. Likewise, the p38MAPK/MEF pathway protects differentiating neurons from cell death during neurogenesis, and in colorectal cancer cells, p38MAPK inhibition causes an autophagic cell death [37,38,39]. Recently, p38MAPK has been also implicated in a survival status known as tumor dormancy, in which tumor cells remain in a quiescent state [40]. Importantly, the downstream effectors mediating p38MAPK pro-survival activity have not yet been fully identified. In MEFs, p38MAPK regulates the Bim/Bcl-xL ratio that may contribute to the survival effects upon H_2_O_2_-induced apoptosis [41]. Consistently, our data also reveals that BMP9 controls Bim expression (and other pro-apoptotic Bcl-2 family members) in a p38MAPK-dependent mechanism, thereby modifying the Bim/Bcl-xL ratio, which may result in cell death.

In the context of a solid tumor, such as the HCC, cells have to deal with an adverse environment, characterized by hypoxia/reoxygenation fluctuations and nutrient deficiency, eventually leading to cell death. Therefore, for a cancer cell, it is essential to develop survival strategies. Based on the premise that p38MAPK may contribute to tumor cell survival and/or metastasis, our data support an essential role for the autocrine BMP9 signaling through p38MAPK as a protective mechanism during lack/shortage of nutrients. Whether or not BMP9-triggered p38MAPK activation plays a similar protective role against hypoxia/reoxygenation injury, also present in the tumor, requires additional studies. Nonetheless, in support of this hypothesis, p38MAPK has been previously involved in the survival effect on hypoxia/anoxia-induced apoptosis in different cellular models [42,43,44].

In conclusion, we have further explored the intracellular mechanisms driving BMP9’s effects in HCC cells. We have shown that BMP9 activates Smad and non-Smad pathways, specifically AKT and p38MAPK cascades. We have demonstrated for the first time that a non-canonical pathway is required for a BMP9-mediated biological effect in liver cancer cells. Significantly, our findings provide new clues for a better understanding of BMP9’s contribution to the hepatocarcinogenic process and push considering BMP9 as a potential target for therapeutic intervention in HCC.

## 4. Experimental Section

### 4.1. Materials

The following reagents were used: BMP9 was from R&D Systems (Minneapolis, MN, USA), and LY294002 (PI3K inhibitor) and SB203580 (p38 inhibitor) were from Calbiochem (La Joya, CA, USA). Dulbecco’s Modified Eagle’s Medium (DMEM) was purchased from LonzaIberica (Barcelona, Spain). Fetal bovine serum (FBS) and trypsin-EDTA were from Gibco-Invitrogen (Barcelona, Spain). Penicillin, streptomycin, Wortmannin (p38 inhibitor), HEPES, bovine serum albumin, propidium iodide and all buffer reagents were from Sigma–Aldrich (Madrid, Spain). [3H]-thymidine (25.0 Curie (Ci)/mmol equivalent to 925 GBq/mmol) and horseradish peroxidase-conjugated secondary antibodies were from GE Healthcare Europe (Barcelona, Spain). Enhanced chemiluminescence (ECL) reagent was from Pierce (Fisher Scientific, Madrid, Spain).

Antibodies against the following proteins were used: rabbit polyclonal antibodies against phospho-Smad1 (Ser463/465)/Smad5 (Ser463/465)/Smad8 (Ser426/428) (#9511), phospho-p38MAPK (Thr180/Tyr1829) (#9211), phospho-AKT (Ser473) (#9271), Puma (#4976) and Smad4 (#9515) were purchased from Cell Signaling Technology (Beverly, MA, USA). Smad2,3 rabbit monoclonal antibody (#610842) and Bim rabbit polyclonal antibody (#559685) were from BD Biosciences (Madrid, Spain). Rabbit polyclonal against p38 MAPK (sc-535) and p27 (c-19) and cyclin D1 mouse monoclonal (DSC-6) antibodies were from Santa Cruz Biotechnology, Inc. (Paso Robles, CA, USA), and PI3K p85 rabbit polyclonal antibody (#06-195) were from Millipore and β-actin (#A5441) from Sigma–Aldrich.

### 4.2. Cell Culture

HepG2, a human HCC epithelial cell line, was obtained from the European Collection of Cell Cultures (ECACC). Cells were grown in DMEM medium supplemented with 10% FBS, 100 U of penicillin and streptomycin per mL and maintained in a humidified incubator at 37 °C and a 5% CO_2_ atmosphere.

### 4.3. Analysis of Cell Number

Proliferation studies were performed as previously described [15]. Briefly, 20,000 cells/well in 12-well plates were plated and serum starved prior to treatment with different factors. Cells were harvested by trypsinization at various time points, and the cell number was determined using a Casy cell counter (Roche, Barcelona, Spain).

### 4.4. Cell Viability Analysis

Viable cells were analyzed by crystal violet staining, as previously described [45]. Cells were incubated with a crystal violet solution (0.2% in 2% ethanol) for 20 min. After that, plates were rinsed with water, allowed to dry and, then, incubated in 1% sodium dodecyl sulfate. The absorbance of each plate was read photometrically at 560 nm.

### 4.5. BrdU Incorporation Assay

A BrdU colorimetric kit from Roche (Barcelona, Spain) was used to determine cell proliferation. Five thousand cells were plated in 96-multiwell plates, starved overnight and treated for 48 h. Cells were labeled with BrdU for 2 h, and incorporation of BrdU into DNA was analyzed following the manufacturer’s instructions, measuring the absorbance of the samples in an ELISA reader at 370 nm.

### 4.6. Thymidine Incorporation Assay

Cells were plated at a density of 17,500 cells/sq cm in DMEM with 10% FBS. The following day, cells were serum starved and incubated for 48 hours with or without BMP9. Incorporation of [3H]-thymidine during the last 40 h of culture was measured in trichloroacetic acid-precipitable material, as previously described [46].

### 4.7. Analysis of Cell DNA Content by Flow Cytometry

Cells were harvested by trypsinization, fixed in 70% ethanol (−20 °C) for 1 minute and treated with RNaseA (10 mg/mL) for 30 min at 37 °C. After propidium iodide staining (0.05 mg/mL, 15 min at room temperature in the dark), cellular DNA content was analyzed in a FACScan flow cytometer (Becton-Dickinson, San Jose, CA, USA). For computer analysis, only signals from single cells were considered (15,000 cells/assay).

### 4.8. Western Blotting

Cell lysates were performed as described previously [15] or using a modified RIPA buffer (5 mM Tris–HCl pH 7.5; 150 mM NaCl; 1% NP-40; 5 mM EGTA; 5 mM EDTA) supplemented with 20 mM phenylmethylsulfonyl fluoride, 1 mM Na orthovanadate, 1 mM phenylmethylsulfonyl fluoride, 1 μg/mL leupeptin and 1 μg/mL aprotinin. Forty to sixty micrograms of protein were separated in 10%–15% acrylamide sodium dodecyl sulfate-polyacrylamide electrophoresis gels and blotted to Immobilon-P membranes (Millipore, Bedford, MA, USA). Western blotting was performed as described previously [15].

### 4.9. siRNA Knockdown Assays

siRNA knockdown assays were performed as previously described [15]. Briefly, HepG2 cells were transfected with a SMARTpool siRNA directed to the human p85α regulatory subunit of PI3K (100 nM) (Reference Number M-003020-04); to human p38α (200 nM) (Reference Number M-003512-06) and their control non-targeting siRNA (100 and 200 nM, respectively) (Dharmacon, Lafayette, CO, USA). siRNA oligonucleotides targeting Smad4 (siSmad4 #1 Reference Number 115650; siSmad4 #2 Reference Number 107685) and its control non-targeting siRNA were purchased from Ambion, Warrington, UK, and used at a 50 nM concentration. Transfection was carried out using oligofectamine transfection reagent from Invitrogen (Barcelona, Spain) according to the manufacturer instructions. Transfected cells were grown for 24 h in complete medium, then trypsinized, diluted to the appropriate cell density and replated in dishes for subsequent assays.

### 4.10. Measurement of Apoptotic Index

The apoptotic index measurement was performed as previously described [47]. After staining with propidium iodide, cells undergoing apoptosis were scored under inverted fluorescence microscope (Eclipse TE300, Nikon, Barcelona, Spain) at high magnification (×60) following standard morphological criteria. Apoptotic indices were calculated after counting a minimum of 1000 cells per treatment in a blinded manner.

### 4.11. Quantitative Reverse Transcriptase-Polymerase Chain Reaction Analysis

After total RNA isolation using the Ribospin Kit (Invitrogen, Barcelona, Spain), RNA yield and purity were analyzed using a spectrophotometer (UV-visible recording spectrophotometer Specord 205, AnalytikJena, Jena, Germany). One microgram of total RNA was reverse-transcribed using the SuperScript III RT kit (Invitrogen). Quantitative PCR was performed using SYBRGREEN (Roche) and the following specific primers: Bax forward 5′-TGCTTCAGGGTTTCATCCAG-3′ and reverse 5′-GGCGGCAATCATCCTCTG-3′; Bcl2 forward 5′-AGGAAGTGAACATTTCGGTGAC-3′ and reverse 5′-GCTCAGTTCCAGGACCAGGC-3′; Bcl2l1 forward 5′-TAGAGTTCCACAAAAGTATC-3′ and reverse 5′-CATGGCAGCAGTAAAGCAAG-3′; Bim forward 5′-AACCACTATCTCAGTG-CAAT-3′ and reverse 5′-GGTCTTCGGCTGCTTGGTAA-3′; Puma forward 5′-GCCCGTGAAG-AGCAAATGAG-3′ and reverse 5′-TGATGAAGGTGAGGCAGGCA-3′; and Gusb 5′-ATCACCGT-CACCACCAGCGT-3′ and reverse 5′-GTCCCATTCGCCACGACTTTGT-3′. For Smad4 detection forward 5′-CTCATGTGATCTATGCCCGTC-3′ and reverse 5′-AGGTGATACAACTCGTTCG-TAGT-3′ and Qiagen commercial primers were used. β-Actin primers for RT-qPCR were obtained from Qiagen. Amplified products were analyzed in an ABI Prism 7900 HT Fast Real-Time (Applied Biosystems, Foster City, CA, USA). The relative amount of target mRNA was determined after normalization against the housekeeping gene (*Gusb*) in each sample.

### 4.12. Statistical Analysis

Statistical analysis was performed by Student’s *t*-test analysis.

## Supplementary Materials

Supplementary materials can be found at http://www.mdpi.com/1422-0067/16/09/20431/s1.
