# Supplementary material for: BMP9-Induced Survival Effect in Liver Tumor Cells Requires p38MAPK Activation
Source: Int J Mol Sci. 2015 Aug 28;16(9):20431–48. doi: 10.3390/ijms160920431 (PMC4613212; doi:10.3390/ijms160920431)
Supplement: Supplementary file 1 [file ijms-16-20431-s001.pdf]

Supplementary Information

Supplementary Materials and Methods

Human recombinant TGF-β was purchased from Calbiochem (La Jolla, CA, USA). [^{3}H]-thymidine (25.0 Ci/mmol) was from GE Healthcare Europe (Barcelona, Spain). Antibodies against the following proteins were used: rabbit polyclonal antibodies against phospho-ATF2 (Thr71) (#9221), phospho-ERK1/2 (Thr202/Tyr204) (#9101) and phospho-Smad2 (Ser465/467) (#3101) were purchased from Cell Signaling Technology (Beverly, MA, USA). Following primers were used for RT-qPCR analysis: Id1 forward: 5′-GGGATTCCACTCGTGTGTTT-3′ and reverse 5′-CTGAGAAGCACCAAACGTGA-3′; Hamp forwars 5′-CACAACAGACGGGACAAC-3′ and reverse 5′-CGCAGCAGA-AAATGCAGA-3′.

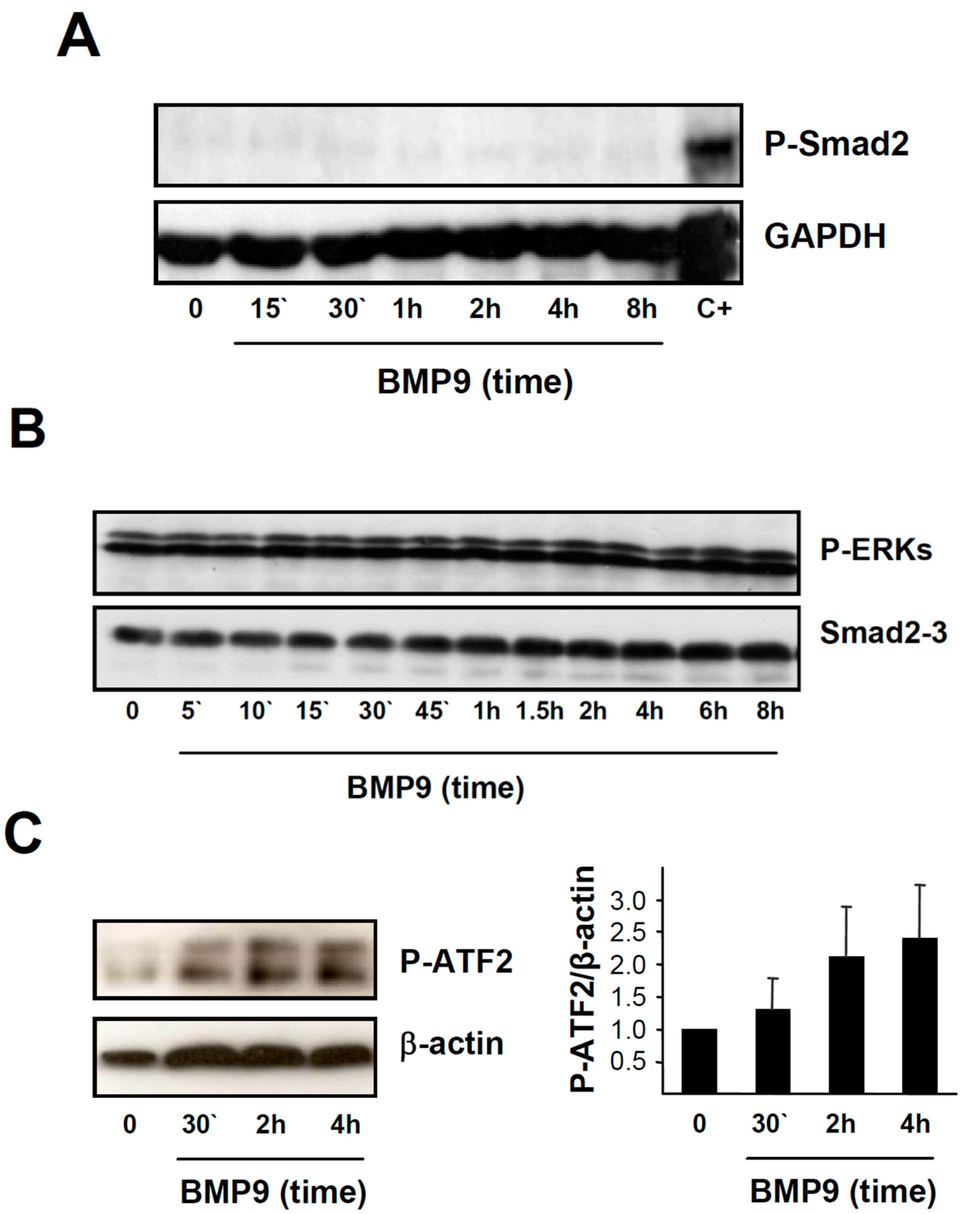

Figure S1. (**A**) HepG2 cells were incubated −/+ BMP9 (5 ng/mL) in 0% FBS media for different periods of time. As positive control of Smad2 phosphorylation (C+), HepG2 cells treated with TGF-β (1 ng/mL) for 1 hour were used. Western blots were performed with antibodies that recognize phospho-Smad2 and GAPDH as loading control. A representative experiment of 2 is shown; (**B**) HepG2 cells were incubated −/+ BMP9 (5 ng/mL) in 0% FBS media for different periods of time. Western blots were performed with antibodies that recognize phospho-ERK and Smad2/3 as loading control. A representative experiment out of 2 is shown; (**C**) HepG2 cells were incubated −/+ BMP9 (5 ng/mL) in 0% FBS media for different periods of time. Western blots were performed with antibodies that recognize phospho-ATF2 and β-actin as loading control. A representative experiment out of 3 is shown (**left** panel). Optical density values relative to loading control were calculated and expressed as fold change relative to untreated, assigned an arbitrary value of 1 (**right** panel).

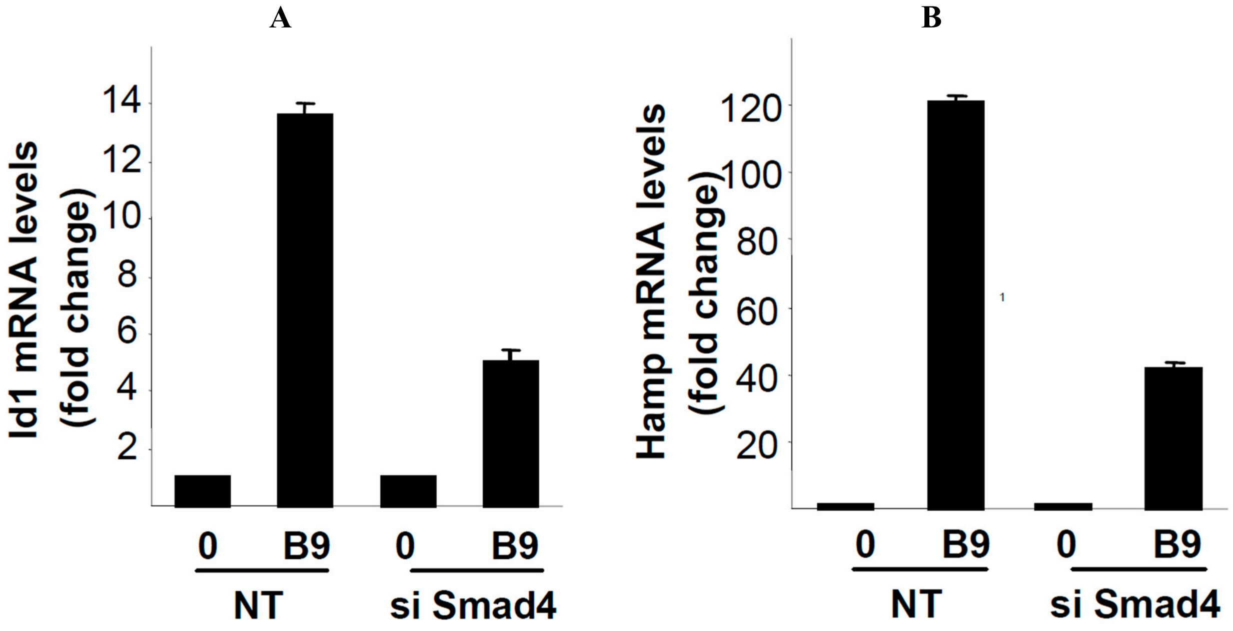

Figure S2. HepG2 cells were transiently transfected with non-targeting negative control siRNA (siRNA NT) or with a Smad4 targeting siRNA #1 (siRNA Smad4). Cells were then serum starved and treated with BMP9 (5 ng/mL) for 24 hours. RNA was isolated and Id1 (**A**) and Hamp (**B**) RNA levels were determined by RT-qPCR and normalized to gusb. Data expressed relative to untreated siRNA NT (assigned an arbitrary value of 1), from three determinations (mean ± S.E.M) from 1 representative experiment out of 3.
